# Evaluation of the early-stage entrepreneurship activity in the United States during the COVID-19 pandemic

**DOI:** 10.3389/fpubh.2022.972203

**Published:** 2022-09-15

**Authors:** Pengsheng Kang, Lin Guo, Zhou Lu, Lili Zhu

**Affiliations:** ^1^Macau University of Science and Technology, Macao, Macao SAR, China; ^2^Tianjin University of Commerce, Tianjin, China; ^3^Qingdao City University, Qingdao, China; ^4^Shenandoah University, Winchester, VA, United States

**Keywords:** COVID-19 pandemic, pandemic, early-stage entrepreneurship activity, startup's early survival rate, entrepreneurship

## Abstract

This paper examines the effects of the COVID-19 pandemic (measured by total cases and deaths per 100K people) on the early-stage entrepreneurship activity (measured by the Kauffman Early-Stage Entrepreneurship indicators) in the United States. The empirical analyses are based on the panel dataset of 51 States between 2020 and 2021. The findings show that the COVID-19 pandemic negatively affects early-stage entrepreneurship activity. Further analyses indicate the positive impact of the COVID-19 pandemic on the startup's early survival rate. However, new entrepreneurs' rate and opportunity share are negatively affected by the COVID-19 pandemic. Implications for the post-COVID-19 era are also discussed.

## Introduction

The COVID-19 pandemic has changed various dimensions of the economic system and has significantly affected various indicators. The COVID-19 pandemic created an external shock, which affected entrepreneurship activities (1). At the begging of the pandemic, the critical target of the policymakers was to decrease the cases of infections and death caused by an unknown virus (2). Different countries' governments have responded to the first wave of lockdown by providing stimulus packages (3). However, the responses have significantly changed across countries since the economic conditions were not the same at the begging of the pandemic (4). For instance, wages were paid in some countries, such as the United Kingdom, but other countries, such as the United States, adopted alternative solutions, such as direct income payment (5). Therefore, implications for the business world and the employees have become necessary during the first wave of lockdown (6, 7).

According to various models, entrepreneurship is the engine of economic growth (8–14). It is the main driving force behind the sustainability force of the free market economies under strong institutions and the rule of law. New inventions provided to potential buyers and firms (sellers) can grow with the market economy (15). Therefore, one of the critical policy implications for the policymakers in free market economies is to sustain the businesses' activities alive (16). Policymakers must provide fertile ground for business activities and open up links for other market economies (17, 18). At this stage, new business opportunities and successful entrepreneurship are the keys to creating new jobs during the COVID-19 era (19–21). However, it is essential that many entrepreneurs' activities were ignored during the second and third waves of lockdowns. Therefore, for various reasons, early-stage entrepreneurship activity, evaluation of entrepreneurial performance, entrepreneurial legitimacy, and entrepreneurial passion are crucial indicators for policymakers.

Given this backdrop, this paper investigates the direct impact of the COVID-19 pandemic (measured by total cases and deaths per 100K people) on the early-stage entrepreneurship activity (measured by the Kauffman Early-Stage Entrepreneurship indicators) in the United States. The empirical analyses are based on the panel dataset of 51 States of the country between 2020 and 2021. Several papers examine the effects of the COVID-19-related shocks on entrepreneurial performance. However, most of these papers have focused on the case of developing countries. In this paper, we focus on the subject of the United States at the state level between 2020 and 2021 to examine the direct impact of the COVID-19 pandemic on early-stage entrepreneurship activity. However, previous papers analyse the effects of the COVID-19 pandemic on economic indicators. To the best of our knowledge, there is no paper in the empirical literature to examine the direct impact of the COVID-19 pandemic on early-stage entrepreneurship activity in the United States. Our paper aims to fill this gap in the literature.

According to the empirical findings, the COVID-19 pandemic negatively affects early-stage entrepreneurship activity in the United States. Further analyses show the positive impact of the COVID-19 pandemic on the startup's early survival rate. However, new entrepreneurs' rate and opportunity share are negatively affected by the COVID-19 pandemic in the United States.

The remaining parts of the paper are organized as follows. Section Literature review provides a brief review of the literature investigating the effects of the COVID-19 pandemic on entrepreneurial performance. Section Data, model and methodology explains the details of the data, the empirical model, and the methodology. Section Empirical results discusses the empirical results. Section Concluding remarks provides the concluding remarks.

## Literature review

Several previous papers focus on the effects of the COVID-19 pandemic on entrepreneurial performance (22, 23). For instance, Lu et al. (24) focus on the effects of the COVID-19 pandemic on small and medium-sized enterprises in China. The authors conducted an online questionnaire and follow-up interviews on 4,807 small and medium-sized enterprises in Sichuan. The authors observe that most firms were negatively affected by disrupted supply chains and declined market demand. These issues created cash-flow risks for various small and medium-sized enterprises in China and negatively affected entrepreneurial performance.

Mu et al. (25) examined the effects of openness on entrepreneurial performance during the COVID-19 pandemic. The paper implements an online questionnaire survey to 238 entrepreneurs of small and micro firms in China from February 18, 2020, to February 26, 2020. The authors find openness increases entrepreneurial performance during the COVID-19 pandemic in related Chinese firms.

Shafi et al. (26) also investigated the role of the COVID-19 pandemic on micro, small, and medium-sized enterprises in Pakistan. The paper creates the data from an online questionnaire survey for 184 firms. The authors observe that most firms are negatively affected by the COVID-19-related shocks. The main problems are lack of credit sources, supply chain disruption, and demand reduction. Most firms go through the wait-and-see policy (over 83% of enterprises), and the authors concluded that the COVID-19 pandemic has negatively affected the entrepreneurial performance in Pakistan. Lu et al. (27) also show that small firms in the United States have been significantly affected by the COVID-19-related shocks. The paper uses the state-level data for the accommodation, food services, hospitality, and leisure sectors from January 10, 2020, to June 24, 2021.

There are also previous papers to analyse the effects of the COVID-19 pandemic on different economic indicators using state-level data in the United States. For instance, Zhang et al. (28) find that employment has been significantly affected by the COVID-19 pandemic at the state level in the United States from January 8, 2020, to May 30, 2020. The results are also valid in the employment of five different sectors. Using the data from January 24, 2020, to June 10, 2020, at the national and state levels, Dong et al. (29) observe that personal consumption expenditures in the United States have been negatively affected by the COVID-19-related shocks.

In short, several previous papers have examined the effects of the COVID-19-related shocks on entrepreneurial performance. However, most of these papers have focused on the case of developing countries, such as China and Pakistan. At this stage, our paper focuses on the case of the United States at the state level. As we have discussed, previous papers analyse the effects of the COVID-19 pandemic on economic indicators. However, there is no paper in the empirical literature to examine the direct impact of the COVID-19 pandemic on early-stage entrepreneurship activity in the United States.

## Data, model and methodology

### Data

This paper focuses on the panel dataset of 51 States in the United States between 2020 and 2021. There are 102 observations in total. Four indicators measure early-stage entrepreneurial activity (30, 31):

1) The rate of new entrepreneurs: This indicator shows the number of new entrepreneurs in a related year. Therefore, it is the widest indicator of the potential for business creation by population.

2) The opportunity share by new entrepreneurs: This indicator is the percentage of new entrepreneurs who created their businesses due to seeing it as an opportunity instead of a necessity. Measuring the number of people who created their businesses as a choice rather than a necessity is essential.

3) The startup's early job creation: This indicator measures the total number of jobs created by start-ups per capita.

4) The startup's early survival rate measures new firms' 1-year average survival rate.

A summary index of entrepreneurship activity is defined as the *Kauffman Early-Stage Entrepreneurship (KESE)* indicator. The KESE indicator measures the early-stage entrepreneurial activity, and the data are obtained from Fairlie (30, 31). The KESE indicator is defined as the equal weights of the four indicators. The Kauffman Early-Stage Entrepreneurship (KESE) indicator is defined as an equally-weighted composite of the four indicators of early entrepreneurship activity.

Each indicator is based on the regional (state) level sample of more than 500,000 observations each year. The data covers more than 5 million employer businesses in the United States, focusing on the United States Census Bureau and Bureau of Labor Statistics (32, 33). Therefore, the KESE indicator follows entrepreneurial activity over the years across different regions (states) within a large longitudinal dataset (30, 31).

We also measure the effects of the COVID-19 pandemic. For this purpose, we use two indicators: The first is the reported total COVID-19 cases, and the second is the total deaths per 100,000 people. These indicators are measured at the state level, and the daily average values in 2020 and 2021 are considered in the panel dataset. The related state-level data in the United States are downloaded from Chetty et al. (34).

A summary of the descriptive statistics is provided in Table 1.

**Table 1:** Descriptive statistics for all states.

**Variable**	**Mean**	**Standard Dev**.	**Min**.	**Max**.	**Observation**
Rate of new entrepreneurs (RNE)	0.003	0.0008	0.001	0.006	102
Opportunity share of new entrepreneurs (OSN)	0.803	0.058	0.651	0.951	102
Startup early job creation (SJC)	4.524	1.073	2.546	7.985	102
Startup early survival rate (SSR)	0.794	0.033	0.628	0.895	102
Kauffman early-stage entrepreneurship index (KESE)	0.261	2.655	−8.086	8.805	102
Total cases per 100K (TC)	6,217	4,997	288	1,5623	102
Total deaths per 100K (TD)	105	81	6	289	102

Data source: Chetty et al. (34) and Fairlie (30, 31).

The rate of new entrepreneurs is an average of 0.003, and the standard deviation of 0.0008. The opportunity share by new entrepreneurs also has an average of 0.803 and a standard deviation of 0.058. The startup's early job creation averages 4.524 and a standard deviation of 1.073. The startup's early survival rate averages 0.794 and has a standard deviation of 0.033. Finally, the KESE indicator has an average of 0.261 with a standard average of 2.655. Regarding the COVID-19 pandemic, the average daily case is 6,217, with a standard deviation of 4,997 across the states. The average daily death number is 105, with a standard deviation of 81.

Table 2 reports the correlation matrix, which shows the correlations between the indicators of early-stage entrepreneurship activity and the COVID-19 pandemic.

**Table 2:** Correlation matrix for all states.

**Indicator**	**RNE**	**OSN**	**SJC**	**SSR**	**KESE**	**TC**	**TD**
Rate of new entrepreneurs (RNE)	1.000	–	–	–	–	–	–
Opportunity share of new entrepreneurs (OSN)	0.167	1.000	–	–	–	–	–
Startup early job creation (SJC)	0.293	0.084	1.000	–	–	–	–
Startup early survival rate (SSR)	−0.063	−0.052	−0.048	1.000	–	–	–
Kauffman early-stage entrepreneurship index (KESE)	0.807	0.437	0.398	−0.407	1.000	–	–
Total cases per 100K (TC)	−0.008	−0.068	−0.060	0.322	−0.124	1.000	–
Total deaths per 100K (TD)	−0.013	−0.216	−0.065	0.299	−0.049	0.877	1.000

Source: Authors' estimations.

Rate of New Entrepreneurs (RNE), Opportunity Share of New Entrepreneurs (OSN), Startup Early Job Creation (SJC), and Kauffman Early-Stage Entrepreneurship Index (KESE) all have positive correlations. As expected, these indicators negatively correlate with the Startup Early Survival Rate (SSR). However, there are mixed correlations between the indicators of early-stage entrepreneurship activity and the COVID-19 pandemic. Rate of New Entrepreneurs (RNE), Opportunity Share of New Entrepreneurs (OSN), Startup Early Job Creation (SJC), and Kauffman Early-Stage Entrepreneurship Index (KESE) negatively correlated with the Total COVID-19 Cases per 100K (TC) and Total COVID-19 Related Deaths per 100K (TD). The COVID-19 indicators positively correlate with the Startup Early Survival Rate (SSR). In addition, two measures of the COVID-19 pandemic, Total COVID-19 Cases per 100K (TC) and Total COVID-19 Related Deaths per 100K (TD), are positively correlated as expected.

### Empirical model and estimation methodology

At this stage, we estimate the following model using fixed effects estimation techniques, the standard econometric methodology in various empirical papers. We consider the robust standard errors clustered at the state level in the fixed effects estimations.


(1) $$\begin{array}{r} ESAA_{it}=\alpha_{0}+\alpha_{1}COVID_{it}+\vartheta_{t}+\mu_{i}+\varepsilon_{it} \end{array}$$

*ESAA*_*it*_ presents the early-stage entrepreneurship activity, which is measured by the Rate of New Entrepreneurs (RNE), Opportunity Share of New Entrepreneurs (OSN), Startup Early Job Creation (SJC), Startup Early Survival Rate (SSR) and the Kauffman Early-Stage Entrepreneurship Index (KESE). *COVID*_*it*_ is the COVID-19-related indicators, which are the Total COVID-19 Cases per 100K (TC) and the Total COVID-19 Related Deaths per 100K (TD). ϑ_*t*_ represents the time-fixed effects in 2020 and 2021. μ_*i*_ Indicates the state-fixed effects. ε_*it*_ represents the error terms in the estimations.

## Empirical results

Table 3 provides state-level early-stage entrepreneurship indicators in the United States in 2020.

**Table 3:** State level early-stage entrepreneurship indicators in the United States in 2020.

**State**	**Rate of new entrepreneurs**	**Opportunity share of new entrepreneurs**	**Startup early job creation**	**Startup early survival rate**	**Kauffman early-stage entrepreneurship (Kese) index**
Alabama	0.0025	0.7987	4.0521	0.7855	−2.0343
Alaska	0.0048	0.7844	3.5307	0.7946	3.0797
Arizona	0.0038	0.8142	4.8657	0.7686	0.9188
Arkansas	0.0033	0.9107	4.1707	0.7714	1.1253
California	0.0043	0.7969	6.3980	0.8149	4.1195
Colorado	0.0035	0.7695	6.5930	0.7800	0.7437
Connecticut	0.0028	0.7448	3.9758	0.8702	1.1113
Delaware	0.0027	0.8529	6.1348	0.7609	−0.6328
District of Columbia	0.0024	0.7719	7.9859	0.7725	−1.2416
Florida	0.0053	0.8572	6.2217	0.7650	5.4653
Georgia	0.0036	0.8396	5.3224	0.7556	0.6489
Hawaii	0.0041	0.8441	3.1779	0.7619	0.9767
Idaho	0.0038	0.8800	6.2919	0.8044	3.8674
Illinois	0.0027	0.7848	4.1516	0.7931	−1.4004
Indiana	0.0025	0.8103	3.4678	0.7783	−2.3225
Iowa	0.0031	0.8312	3.3973	0.7971	0.0949
Kansas	0.0030	0.8947	3.9341	0.7547	−0.5803
Kentucky	0.0027	0.7945	3.6588	0.7885	−1.6211
Louisiana	0.0037	0.7693	4.2002	0.8025	0.9372
Maine	0.0040	0.8556	4.4367	0.7833	2.3477
Maryland	0.0026	0.7929	3.9328	0.7649	−2.7217
Massachusetts	0.0027	0.6597	5.0531	0.8033	−2.5546
Michigan	0.0029	0.7430	4.1285	0.7704	−2.4118
Minnesota	0.0018	0.6647	3.5717	0.8067	−4.9027
Mississippi	0.0032	0.8387	3.8054	0.7934	0.2947
Missouri	0.0037	0.7902	4.9635	0.7480	−0.3946
Montana	0.0035	0.7815	5.4788	0.8084	1.5446
Nebraska	0.0027	0.8238	4.8581	0.7962	−0.3706
Nevada	0.0032	0.7991	5.3043	0.7552	−1.0495
New Hampshire	0.0031	0.8273	3.5831	0.7682	−1.0303
New Jersey	0.0036	0.7984	6.2993	0.7931	1.7584
New Mexico	0.0051	0.8075	4.0868	0.7972	4.3910
New York	0.0039	0.8388	4.9857	0.7629	1.4155
North Carolina	0.0031	0.8040	4.9010	0.7745	−0.5621
North Dakota	0.0032	0.9512	4.3776	0.7842	1.9200
Ohio	0.0025	0.7339	3.7249	0.7876	−2.9550
Oklahoma	0.0044	0.8390	5.6188	0.7878	3.6147
Oregon	0.0029	0.8572	5.0302	0.8631	3.2502
Pennsylvania	0.0018	0.8309	3.6171	0.7892	−3.0198
Rhode Island	0.0016	0.8071	3.5923	0.7585	−5.1373
South Carolina	0.0026	0.8525	5.4242	0.7733	−0.8227
South Dakota	0.0029	0.8297	4.2442	0.7723	−1.1091
Tennessee	0.0035	0.8802	4.5755	0.8337	3.4338
Texas	0.0038	0.7963	5.5792	0.7940	1.9849
Utah	0.0024	0.8602	5.2979	0.7667	−1.5018
Vermont	0.0040	0.7917	2.9681	0.7811	0.7779
Virginia	0.0023	0.8009	5.1322	0.7609	−2.7185
Washington	0.0027	0.7397	4.5496	0.6287	−8.0868
West Virginia	0.0016	0.8531	3.2289	0.8953	0.6977
Wisconsin	0.0021	0.8335	3.5003	0.7881	−2.3543
Wyoming	0.0040	0.8799	5.7010	0.7685	2.8178

Data source: Fairlie (30, 31).

According to the results in Table 3, the level of the Kauffman Early-Stage Entrepreneurship (KESE) indicator has the highest level in Florida (5.465), New Mexico (4.391), and California (4.119), respectively. The lowest values are observed in Washington (−8.086) and Rhode Island (−5.137). Florida and New Mexico are the top states in the Rate of New Entrepreneurs (0.0053 and 0.0051), respectively. Opportunity Share of New Entrepreneurs has the highest scores in North Dakota and Arkansas. Startup Early Job Creation scores highest in the District of Columbia and Colorado. Finally, Startup Early Survival Rate has the largest value in West Virginia and Connecticut, respectively.

Table 4 reports state-level early-stage entrepreneurship indicators in the United States in 2021.

**Table 4:** State level early-stage entrepreneurship indicators in the United States in 2021.

**State**	**Rate of new entrepreneurs**	**Opportunity share of new entrepreneurs**	**Startup early job creation**	**Startup early survival rate**	**Kauffman early-stage entrepreneurship (Kese) index**
Alabama	0.0026	0.7722	3.4588	0.7795	−2.5810
Alaska	0.0042	0.7761	3.5555	0.8027	1.9035
Arizona	0.0039	0.7843	4.7146	0.8165	2.4049
Arkansas	0.0035	0.9306	3.9182	0.8054	2.8989
California	0.0043	0.7757	5.7297	0.8256	4.0257
Colorado	0.0042	0.7259	6.0851	0.8195	2.9168
Connecticut	0.0031	0.6938	3.9780	0.8129	−1.1055
Delaware	0.0026	0.7999	4.7410	0.8231	−0.0149
District of Columbia	0.0022	0.7662	6.4622	0.7506	−3.2868
Florida	0.0061	0.8608	6.5273	0.8049	8.8057
Georgia	0.0047	0.8156	5.7386	0.7981	4.3765
Hawaii	0.0035	0.7983	3.0252	0.7341	−2.1592
Idaho	0.0033	0.8933	6.1123	0.8085	3.0410
Illinois	0.0027	0.7372	4.3184	0.8480	0.1600
Indiana	0.0023	0.7633	3.8109	0.8359	−1.0486
Iowa	0.0022	0.8688	2.8399	0.8375	−0.1085
Kansas	0.0028	0.8635	3.9048	0.7679	−1.1004
Kentucky	0.0029	0.7229	3.2233	0.8013	−1.8391
Louisiana	0.0037	0.8254	4.0938	0.8000	1.6110
Maine	0.0042	0.7911	4.3411	0.8292	3.4212
Maryland	0.0029	0.8072	2.6637	0.8121	−0.5115
Massachusetts	0.0027	0.6874	4.4657	0.8209	−1.6042
Michigan	0.0029	0.6512	3.5882	0.7893	−3.2415
Minnesota	0.0020	0.7630	3.4212	0.8204	−2.5648
Mississippi	0.0037	0.8194	3.4065	0.8243	2.2428
Missouri	0.0037	0.8166	4.7426	0.7712	0.8169
Montana	0.0036	0.7576	6.1407	0.8104	1.7081
Nebraska	0.0028	0.7754	4.8399	0.7639	−2.1321
Nevada	0.0034	0.7636	6.0649	0.8321	2.2196
New Hampshire	0.0029	0.7227	3.7092	0.7700	−2.9583
New Jersey	0.0037	0.7227	5.8782	0.7995	0.9987
New Mexico	0.0055	0.8309	3.3012	0.7758	4.4450
New York	0.0038	0.8186	4.0834	0.7924	1.4865
North Carolina	0.0034	0.7647	5.7844	0.8274	1.9352
North Dakota	0.0029	0.9129	4.2145	0.7825	0.5884
Ohio	0.0028	0.7377	3.6897	0.8139	−1.3676
Oklahoma	0.0044	0.8456	5.6800	0.8231	5.0189
Oregon	0.0034	0.7661	4.9315	0.7838	−0.2053
Pennsylvania	0.0017	0.7791	3.4309	0.8333	−2.5525
Rhode Island	0.0019	0.6694	3.5368	0.7714	−6.0358
South Carolina	0.0029	0.8400	3.9249	0.8234	0.9551
South Dakota	0.0024	0.8474	3.9213	0.8099	−0.5856
Tennessee	0.0035	0.8114	4.5577	0.8072	1.4072
Texas	0.0037	0.7957	5.1833	0.8190	2.4699
Utah	0.0025	0.9140	6.0240	0.8183	1.7987
Vermont	0.0042	0.7517	2.5466	0.7854	0.5605
Virginia	0.0026	0.7989	4.5891	0.7954	−1.1570
Washington	0.0029	0.7567	4.4610	0.8917	2.5975
West Virginia	0.0017	0.8228	3.4415	0.8108	−2.7846
Wisconsin	0.0022	0.8524	3.6937	0.8235	−0.6371
Wyoming	0.0041	0.8518	3.8752	0.7656	1.6708

Data source: Fairlie (30, 31).

According to the findings in Table 4, the level of the Kauffman Early-Stage Entrepreneurship (KESE) indicator has the highest value in Florida (8.805), Oklahoma (5.019), and New Mexico (4.445), respectively. The lowest values are in Rhode Island (−6.035) and the District of Columbia (−3.286). Florida and New Mexico are again the top states in the Rate of New Entrepreneurs (0.0061 and 0.0055), respectively. The Opportunity Share of New Entrepreneurs has the highest scores in Arkansas and Utah. The Startup Early Job Creation scores the highest in Florida and the District of Columbia. Finally, Startup Early Survival Rate has the largest value in Washington and Illinois.

It seems that Florida has been the state with the highest value in terms of the Kauffman Early-Stage Entrepreneurship (KESE) indicator. New Mexico maintained a good score from 2020 to 2021. Rhode Island has the lowest score both in 2020 and 2021. Some states, such as Washington, gained a place from 2020 to 2021, but California seemed to be lost place between 2020 and 2021.

Table 5 provides the results for the COVID-19 Related Indicators in the United States in 2020 and 2021.

**Table 5:** The COVID-19 related indicators in the United States in 2020 and 2021.

**State**	**Total cases per 100K (2020)**	**Total cases per 100K (2021)**	**Total deaths per 100K (2020)**	**Total deaths per 100K (2021)**
Alabama	2,163	12,639	36	240
Alaska	1,258	11,844	6	60
Arizona	1,838	13,095	41	246
Arkansas	1,986	13,052	31	213
California	1,245	10,310	22	155
Colorado	1,291	10,066	30	124
Connecticut	1,564	9,678	103	228
Delaware	1,730	11,582	50	175
District of Columbia	1,681	7,289	68	156
Florida	2,042	12,321	39	194
Georgia	1,863	11,457	41	200
Hawaii	485	3,448	6	43
Idaho	1,940	12,068	20	137
Illinois	1,824	11,339	50	200
Indiana	1,699	12,105	44	208
Iowa	2,378	12,620	34	193
Kansas	1,749	12,020	21	182
Kentucky	1,339	11,951	21	164
Louisiana	2,468	12,038	84	247
Maine	374	5,399	9	66
Maryland	1,552	7,722	49	154
Massachusetts	1,573	10,391	95	255
Michigan	1,367	9,961	61	203
Minnesota	1,687	11,242	30	136
Mississippi	2,255	12,511	63	266
Missouri	1642	11,217	28	171
Montana	1,516	11,917	18	166
Nebraska	2,052	12,266	21	131
Nevada	1,894	11,603	33	194
New Hampshire	626	7,684	24	100
New Jersey	2,080	11,340	140	289
New Mexico	1,477	10,643	34	205
New York	2,074	6,833	138	207
North Carolina	1,402	10,509	22	132
North Dakota	2,840	15,623	37	210
Ohio	1,229	10,280	30	170
Oklahoma	1,650	12,730	19	183
Oregon	616	5,705	10	74
Pennsylvania	1,178	9,695	51	213
Rhode Island	2,189	14,469	79	251
South Carolina	1,834	12,785	39	201
South Dakota	2,636	14,758	31	229
Tennessee	2,044	13,886	25	191
Texas	1,649	11,212	30	188
Utah	1,925	13,806	11	77
Vermont	288	4,366	9	44
Virginia	1,243	8,395	26	130
Washington	805	6,607	19	84
West Virginia	853	10,368	15	175
Wisconsin	1,892	12,495	21	139
Wyoming	1,436	12,438	11	148

Data source: Chetty et al. (34).

Table 5 shows North Dakota, South Dakota, and Louisiana have the greatest values for the total COVID-19 cases per 100K people in 2020. Interestingly, these findings did not change significantly in 2021, as the largest values for the total COVID-19 cases per 100K people were observed in North Dakota, South Dakota, and Rhode Island in 2021. In addition, New Jersey, New York, and Connecticut have the biggest values for the total COVID-19-related deaths per 100K people in 2020. Interestingly, this evidence slightly changed in 2021 as the greatest values for the total COVID-19-related deaths per 100K people were observed in New Jersey, Mississippi, and Massachusetts in 2021. Note that COVID-19 vaccines have been fully effective in 2021, and there is a significant change in the randomness of the virus spread.

Table 6 also reports the findings of the fixed effects estimations with the robust standard errors clustered at the state level.

**Table 6:** Fixed effects estimations for the effects of the COVID-19 pandemic on early-stage entrepreneurship indicators.

**Indicators**	**RNE**	**RNE**	**OSN**	**OSN**	**SJC**	**SJC**	**SSR**	**SSR**	**KESE**	**KESE**
Total Cases per 100K	−0.734 (0.524)	–	−0.207^***^ (0.067)	–	−0.231^***^ (0.086)	–	0.220^***^ (0.056)	–	−0.573^**^ (0.285)	–
Total Deaths per 100K	–	−0.567 (0.350)	–	−0.139^***^ (0.042)	–	−0.168^***^ (0.054)	–	0.141^***^ (0.036)	–	−0.371^**^ (0.175)
Countries	102	102	102	102	102	102	102	102	102	102
R-squared (adjusted)	0.039	0.051	0.163	0.164	0.125	0.146	0.157	0.141	0.054	0.050

The dependent variables are five early-stage entrepreneurship indicators.

The constant terms are included. The robust standard errors are reported in parentheses.

*** p < 0.01

and

** p < 0.05.

Source: Authors' estimations.

According to the findings in Table 6, both total cases per 100K people and total COVID-19-related deaths per 100K people have significantly and negatively affected the Opportunity Share of New Entrepreneurs (OSN), the Startup Early Job Creation (SJC), the and the Kauffman Early-Stage Entrepreneurship Index (KESE). The related coefficients of the total cases per 100K people and total COVID-19-related deaths are significant at the 1% level for the Opportunity Share of New Entrepreneurs (OSN), the Startup Early Job Creation (SJC), the Startup Early Survival Rate (SSR). At the same time, they are statistically significant at the 5% level for the Kauffman Early-Stage Entrepreneurship Index (KESE). It is important to note that the effects of the total cases per 100K people and total COVID-19-related deaths on the Rate of New Entrepreneurs (RNE) are adverse, but the coefficients are statistically insignificant.

The effects of the total cases per 100K people and total COVID-19-related deaths on the Startup Early Survival Rate (SSR) are positive. The related coefficients are statistically significant at the 1% level. Finally, the Adjusted R-squared scores change from 0.039 to 0.164.

## Concluding remarks

In this paper, we examined the effects of the COVID-19 pandemic, which is measured by total cases and deaths per 100K people on the early-stage entrepreneurship activity, measured by the Kauffman Early-Stage Entrepreneurship indicators in the United States. The empirical analyses are based on the panel dataset of 51 States from 2020 to 2021. It has been found that the COVID-19 pandemic has negatively affected early-stage entrepreneurship activity. Further empirical analyses showed the positive impact of the COVID-19 pandemic on the startup's early survival rate. However, new entrepreneurs' rate and opportunity share are negatively affected by the COVID-19 pandemic.

Overall, our paper shows the adverse effects of the COVID-19 pandemic on the Kauffman Early-Stage Entrepreneurship indicators. However, our findings are limited to the United States economy. Future articles can focus on other developing and developed economies, where the early-stage entrepreneurship activity data are available. We suggest that the case of China and the United Kingdom can be notable countries to investigate the possible effects of the COVID-19-related uncertainty indicators on early-stage entrepreneurship activity.

## Data availability statement

The original contributions presented in the study are included in the article/supplementary material, further inquiries can be directed to the corresponding author.

## Author contributions

PK and LG: conceptualization and methodology. PK and LZ: formal analysis. PK and LG: writing—original draft. ZL and LZ: writing-revision. ZL: funding acquisition and project management. All authors contributed to the article and approved the submitted version.

## Funding

The authors acknowledge financial support from the Tianjin Social Science Program (Award #: TJYJ20-012).

## Conflict of interest

The authors declare that the research was conducted in the absence of any commercial or financial relationships that could be construed as a potential conflict of interest.

## Publisher's note

All claims expressed in this article are solely those of the authors and do not necessarily represent those of their affiliated organizations, or those of the publisher, the editors and the reviewers. Any product that may be evaluated in this article, or claim that may be made by its manufacturer, is not guaranteed or endorsed by the publisher.
